# Does second language decrease cyber deception? Cyber contextual organization of language and deception framework

**DOI:** 10.3389/fpsyg.2026.1923639

**Published:** 2026-10-06

**Authors:** Chen Zhang, HongYu Wang

**Affiliations:** 1 School of Teacher Education, Xuzhou University of Technology, Xuzhou, China; 2 School of Education and Psychology, Shaoxing University, Shaoxing, China

**Keywords:** cyber contextual organization of language and deception framework, cyber deception, foreign language effect, second language, social norms

## Abstract

**Introduction:**

Cyber deception is common in online communication, yet whether using a foreign (second) language increases or decreases dishonest behavior remains debated. We propose the Cyber Contextual Organization of Language and Deception (CCOLD) framework, which integrates psychological dynamics (language), practical goals (lie type), and communication conventions (social norms) to predict cyber deception.

**Methods:**

In three online experiments, participants were randomly assigned to a native-language (Chinese) or foreign-language (English) condition and judged deception scenarios that varied in lie type (black lie vs. white lie in Experiment 1; white lie vs. truth in Experiment 2; black lie vs. truth in Experiment 3) and social norm (descriptive vs. subjective). Data were analyzed with linear mixed-effects models controlling for age, gender, language background, and self-reported English proficiency.

**Results:**

The foreign-language effect was not uniform. Experiment 1 showed a language and lie interaction, such that foreign-language use increased the acceptability of black lies but not white lies. Experiments 2 and 3 showed three-way interactions: under subjective norms, foreign-language use reduced white-lie (Experiment 2) and black-lie (Experiment 3) willingness while increasing truth willingness, whereas language effects were not significant under descriptive norms.

**Discussion:**

The effect of foreign language on cyber deception is conditional rather than fixed, jointly depending on lie type and social-norm type. The CCOLD framework accounts for these boundary conditions and offers implications for online ethical education and deception prevention.

## 1 Introduction

### 1.1 Cyber deception in text-based online contexts

With the advancement of internet technology, the internet has permeated every aspect of life, work, and study (Ayub and Ashraf, 2025; Drouin, 2016; Gibbs, 2025; Robert et al., 2026). However, the internet is an online platform where goodwill and malice coexist, potentially filled with both truth and lies simultaneously (Morgan and Fowers, 2022). Cyber deception is a form of negative online behavior. Caspi and Gorsky (2006) found that over 30% of individuals admitted to having engaged in online deception, and that frequent internet users, younger users, and more technologically adept users were more inclined to perpetrate cyber deception. Cartwright and Xue (2023) found that people exhibit higher levels of dishonesty when using smartphones compared to using pen and paper.

Cyber deception refers to the behavioral pattern in which individuals, for certain purposes, proactively convey and exchange false information to their interaction partners during online interactive communication (George et al., 2016; McArthur et al., 2023; Zhang et al., 2024). In the process of interpersonal interaction, compared with verbal cues, non-verbal cues play a very important role in strategic interactions with strangers. However, given the virtual and anonymous nature of the online environment, compared with face-to-face interaction environments, online interpersonal interactions often lack some non-verbal cues (such as facial cues and vocal cues) (Čekić, 2025; Gogollari et al., 2026; Őrsi et al., 2026; Scott et al., 2022), compelling individuals to rely more heavily on textual cues on social media to judge the veracity of information. Therefore, this study selects textual content in online interpersonal interactions as the focus of cyber deception research.

### 1.2 Foreign language effect on moral decision-making

In modern online life, the use of a foreign language is common; especially when communicating with netizens globally via QQ/WeChat (Chen and Lu, 2026), individuals may enter a foreign language usage environment at any time (Dai, 2026). Therefore, it is imperative to deeply understand the relationship between second language and cyber deception. The Foreign Language Effect (FLE) (Almanea, 2026; Samuel and Boeckle, 2026; Xie et al., 2026) refers to the notion that compared to a native language, people’s judgments and decisions in a foreign language may differ slightly (Hu and Navarrete, 2024; Keysar et al., 2012).

Prior research focusing on the association between second language and deception decision-making has yielded inconsistent results. Non-significant results suggest that the effect of a foreign language on lying behavior is not identified (Alempaki et al., 2021), or that there is no interaction between deceptive language and second language processing (Duñabeitia and Costa, 2015; Suchotzki and Gamer, 2018). Significant results may fall into two aspects: on one hand, a second language positively predicts unethical decision-making. For example, using a foreign language increases selfish lying behavior (Gai and Puntoni, 2021). On the other hand, a second language negatively predicts unethical decision-making (Bereby-Meyer et al., 2020). For instance, when individuals use a second language instead of their native language, the proportion of false reports is lower, indicating fewer dishonest responses (Bereby-Meyer et al., 2020); participants using a foreign language in moral contexts are more inclined to accept harm to maximize outcomes and demonstrate lower risk aversion in decision-making contexts (Circi et al., 2021).

### 1.3 The role of deception motivation: black lies, white lies, and truth

The motivational type of a lie, such as self-interest, altruism, or no apparent motive, is a key variable affecting the language effect. Focusing on black lies (self-interested deception), Bereby-Meyer et al. (2020), using a dice-rolling paradigm across four different native language backgrounds, found that using a foreign language (English) reduced the rate of black lies. Self-interested lies are intuitive impulses, and the non-automaticity of foreign language processing enhances deliberative control, thereby promoting honesty. However, Suchotzki and Gamer (2018), in a context closer to black lies, found that the difference in reaction time between lying and truth-telling under the foreign language (English) condition was reduced compared to the native language (German) condition, but this was mainly due to prolonged reaction times for truth-telling. Thus, the foreign language primarily interfered with truth-telling rather than promoting lying. Although both studies examined self-interested lies, they yielded behavioral effects in different directions (foreign language reduces vs. foreign language does not reduce black lies).

Unlike the aforementioned studies, Gai and Puntoni (2021) differentiated between black lies and white lies, demonstrating that the direction of the FLE depends on the lie’s motivation. Based on self-diagnosticity theory, a foreign language, by reducing the diagnosticity of behavior for one’s self-concept, increases black lies (a dishonest self) due to reduced self-threat and decreases white lies (a compassionate self) due to reduced self-affirmation. This framework effectively explains the apparent contradictions and suggests that the internal heterogeneity of black lies (e.g., moral justifiability) can affect whether a foreign language facilitates deception. Furthermore, Duñabeitia and Costa (2015) examined experimental lies without direct benefit motives (truthfully/falsely naming animal pictures) and found that lying and foreign language use only produced additive cognitive costs (pupil dilation, reaction time) without interaction, indicating that when lies lack genuine tempting motivation, language and deception may only manifest as a linear superposition of cognitive load. In summary, deception motivation (self-interest, altruism, no motive) is a key dimension for understanding the relationship between foreign language and lying.

### 1.4 Social norms as boundary conditions

An aspect of the foreign language’s influence on cyber deception that awaits exploration pertains to the norms of online deception (Göckeritz et al., 2010; Köbis et al., 2015; Mitra and Shahriar, 2020; Raihani and McAuliffe, 2014; Zhao et al., 2019). Social norms are shared standards of socially acceptable behavior within a social group regarding specific social situations, where violating these standards incurs social consequences (Cialdini and Goldstein, 2004; Hu and Navarrete, 2024). Among social norms, descriptive norms relate to attitudes and beliefs about behavioral prevalence, whereas subjective norms relate to perceptions of social expectations (Costenbader et al., 2017; Morris et al., 2015). In this paper, we refer to social norms concerning the perception of third-person behavior as descriptive norms, and norms concerning first-person behavioral standards as subjective norms.

Currently, there is limited research on the relationship between the FLE of cyber deception and social norms. Alempaki et al. (2021) noted that in a Chinese sample, both social norms and personal norms under the native language (Chinese) condition were stricter than under the foreign language (English) condition. The researchers found that a foreign language significantly reduced the proportion of lying in the Chinese sample. Hu and Navarrete (2024) comparing native language (Italian) and foreign language (English), found that using a foreign language could reduce the influence of descriptive norms.

### 1.5 The present study and research questions

Based on existing research, there are two aspects worthy of further in-depth exploration: (1) The conclusions of related research are highly controversial, and a systematic theoretical explanation framework has not yet formed. Existing studies have noted the influence of second language factors, motivational factors, and normative factors, but no systematic theoretical explanation framework exists for the deep-seated causes of belief formation regarding cyber deception. In particular, few studies have explained the reasons for the generation of cyber deception from the perspective reflecting the essential question of “how psychological dynamics, practical goals, and deception context systematically influence cyber deception.”

(2) Existing studies still have notable limitations. Hu and Navarrete (2024) concerning descriptive norms about deception should be considered more as descriptive norms about deception detection. In other words, Hu and Navarrete (2024) confused “most people can detect lies” (descriptive norm about lie detection) with “most people will lie” (descriptive norm about lying). If subjective norms are related to lying, while descriptive norms are related to lie detection, then there is no reason for comparing these two types of norms.

Based on this, the present research, through quantitative analysis, aims to tackle three major challenges in current cyber deception prevention: (1) How does a second language influence the generation of cyber deception? (2) How do black lies and white lies influence the generation of cyber deception? (3) How do descriptive norms and subjective norms influence the generation of cyber deception?

To address the above issues in depth, this study, from the perspective of the occurrence of cyber deception, utilizes the logical framework of the Cyber contextual organization of language and deception (CCOLD) framework. It explains the psychological mechanisms influencing the occurrence of cyber deception, along with the theoretical logic and operational mechanisms behind its influencing factors, from three levels: psychological dynamics, practical goals, and communication norms. Behavioral experiments are then employed to conduct empirical analysis of the theoretical hypotheses, aiming to provide precise strategies for preventing online fraud. This study was preregistered at https://doi.org/10.17605/OSF.IO/2RUVS.

## 2 The CCOLD framework and hypotheses

### 2.1 Cyber contextual organization of language and deception

The CCOLD framework adapted from the contextual organization of language and deception (COLD) framework, which was proposed by Markowitz and Hancock (2019). Unlike the single pursuit of traditional deception research, COLD theory emphasizes that for any deceptive act, there exist three interconnected core dimensions: psychological dynamics, practical goals of the speaker, and communication conventions of deception occurrence. The COLD provides a more comprehensive and dynamic analytical framework for understanding deceptive behavior by integrating contextual factors such as psychological dynamics, practical goals, and genre conventions. This COLD is expected to play a greater role in fields such as deception detection, communication strategy design, and interpersonal interaction (Li et al., 2026; Markowitz, 2024; Markowitz et al., 2023; Markowitz, 2026).

Based on the COLD, this study proposes a contextual deception theory framework applicable to the online environment. CCOLD situates the occurrence of deceptive acts during interpersonal interaction within online social media. This study regards second language as one of the psychological dynamic factors, black lies and white lies as the speaker’s deception goals, and social norms as representing the communication norms of deception. Overall, this study expects to use empirical research to verify the applicability and interpretability of the CCOLD framework in deception research.

### 2.2 Three Core dimensions of CCOLD

#### 2.2.1 Psychological dynamics

Psychological dynamics refer to the emotional and cognitive states involved in deception decision-making. In this study, the native versus foreign language condition is the central psychological-dynamics manipulation because foreign-language processing is associated with reduced emotional reactivity and increased deliberative processing (Keysar et al., 2012; Hu and Navarrete, 2024; Circi et al., 2021). This dimension is expected to change the diagnosticity of deception for the self and to alter the salience of moral self-threat.

#### 2.2.2 Practical goals

Practical goals refer to what the speaker attempts to achieve through deception. Black lies serve self-interested goals and are typically regarded as more morally problematic, whereas white lies serve other-oriented, prosocial goals and are viewed more positively (Cantarero et al., 2018; Lu et al., 2019). Truth-telling represents honest disclosure. Across the three experiments, this dimension is operationalized as black lie versus white lie, white lie versus truth, and black lie versus truth, respectively.

#### 2.2.3 Communication conventions

Communication conventions refer to the social rules and online settings through which deception is communicated. In this study, descriptive norms represent perceptions of what others do, whereas subjective norms represent perceptions of what the individual should do (Cialdini and Goldstein, 2004; Alempaki et al., 2021; Hu and Navarrete, 2024). The text-only online platform further reduces non-verbal cues and makes the distinction between descriptive and subjective norms especially relevant to cyber deception.

### 2.3 From the CCOLD framework to hypotheses

Experiment 1 tests the interaction between psychological dynamics and practical goals, with communication conventions examined as descriptive versus subjective norms.

Experiment 2 tests the interaction between psychological dynamics and practical goals using the comparison of white lies and truth, while communication conventions are again represented by descriptive versus subjective norms.

Experiment 3 tests the interaction between psychological dynamics and practical goals using the comparison of black lies and truth, with communication conventions represented by descriptive versus subjective norms.

#### 2.3.1 Hypotheses for experiment 1

First, existing research has found contradictory effects of foreign language on moral decisions: Based on Interpersonal Deception Theory (IDT) (AbouGrad and Riaz, 2026; Buller and Burgoon, 1996; Hafeez and Naveed, 2026), information transmission in interpersonal deception may depend on the communicator’s goals (e.g., self-interest or altruism). Some studies show foreign language increases self-interested deception (e.g., Gai and Puntoni, 2021), while others find foreign language prompts individuals to accept harm (Circi et al., 2021). This divergence may stem from not distinguishing between types of deception (e.g., black lies/white lies). Cyber deception, characterized by anonymity and a lack of non-verbal cues, may amplify foreign language’s inhibitory effect on emotional processing (Hu and Navarrete, 2024). White lies (e.g., to avoid hurting others) usually accompany positive moral evaluations (Cantarero et al., 2018), whereas black lies (e.g., for personal gain) trigger stronger moral conflicts. The emotional blunting effect of foreign language (Hu and Navarrete, 2024) may primarily alleviate negative emotions associated with black lies. Therefore, we propose:

*H1a*: The acceptability of cyber deception occurrence is significantly higher when using a foreign language (English) than when using a native language (Chinese).

*H1b*: This effect is stronger for black lies (self-interested deception) than for white lies (altruistic deception).

Second, Hu and Navarrete (2024) confirmed that foreign language primarily weakens descriptive norms, not subjective norms. This is consistent with the finding of Alempaki et al. (2021): foreign language reduces Chinese speakers’ judgments on the severity of lying. Foreign language may reduce the perceived likelihood of cyber deception occurrence by weakening the influence of descriptive norms. Therefore, we propose:

*H1c*: Descriptive norms reduce the evaluation of cyber deception occurrence under the foreign language condition, while subjective norms remain stable.

Third, generally speaking, black lies are perceived as immoral, while white lies are perceived as more moral. Combined with the moral attribute differences of lie types, the weakening effect of foreign language on cyber deception is most significant for black lies under the descriptive norm condition. Therefore, we propose: a three-way interaction among language type, lie type, and social norm.

*H1d*: Under the descriptive norm condition, foreign language significantly reduces the likelihood of black lies occurring (vs. native language); there is no significant foreign language effect under other conditions (subjective norm/white lies).

#### 2.3.2 Hypotheses for experiment 2

Since there are still many differences between black lies and white lies, such as different backgrounds, different beneficiaries, and different interpersonal relationships, Experiment 2, building on Experiment 1, further explores the comparison between white lies and honest truth-telling under the same story background and with the same beneficiary of the lie. It aims to investigate whether and how foreign language affects individuals’ evaluations and choices between benevolent deception and truth.

*H2a*: The acceptability of white lies and truth is significantly higher when using a foreign language (English) than when using a native language (Chinese).

*H2b*: This effect is more significant for the truth and not significant for white lies.

*H2c*: Descriptive norms reduce the evaluation of cyber deception occurrence under the foreign language condition, while subjective norms remain stable.

*H2d*: Under the descriptive norm condition, foreign language significantly reduces the likelihood of truth occurrence (vs. native language); there is no significant foreign language effect under other conditions (subjective norm/white lies).

#### 2.3.3 Hypotheses for experiment 3

Experiment 3 explores the direct comparison between black lies and truth under the same story background and with the same beneficiary of the lie. It aims to clearly reveal the role of second language use in the trade-off between black lying behavior and truth-telling, and to test the pathway of social norms.

*H3a*: The acceptability of black lies and truth is significantly higher when using a foreign language (English) than when using a native language (Chinese).

*H3b*: This effect is more significant for black lies and not significant for the truth.

*H3c*: Descriptive norms reduce the evaluation of cyber deception occurrence under the foreign language condition, while subjective norms remain stable.

*H3d*: Under the descriptive norm condition, foreign language significantly reduces the likelihood of black lies occurring (vs. native language); there is no significant foreign language effect under other conditions (subjective norm/truth).

## 3 Empirical research

### 3.1 Experiment 1: how second language influences cyber deception: black lies and white lies

#### 3.1.1 Research purpose

Experiment 1 explores the influence of second language (English) use on cyber deception, i.e., the foreign language effect (FLE) of cyber deception, and examines the roles of lie type (black lie/white lie) and social norm (descriptive norm/subjective norm). Using a mixed experimental design, participants were randomly assigned to either native (Chinese) or foreign (English) language conditions and were asked to assess the likelihood of deception occurring in online deception scenarios.

#### 3.1.2 Participants

Experiment 1 used *a priori* power analysis in G*Power (Faul et al., 2007) to estimate the sample size, selecting a small to medium effect size f^2^ = 0.02 (Cantarero et al., 2018; Hu and Navarrete, 2024), α = 0.05, and power = 0.95, yielding a required sample size of 108. Therefore, 120 participants (*N* = 120, 54 males and 66 females) were planned to be recruited from an online platform, with 60 participants randomly assigned to the native language group and the other 60 participants randomly assigned to the English group. The average age of participants was 21.05 years old (SD = 1.321, range = 18–24).

#### 3.1.3 Materials

We selected and adapted 10 black lie scenarios and 10 white lie scenarios from previous studies (Cantarero et al., 2018; Hu and Navarrete, 2024; Moreno et al., 2016). In these scenarios, a person lies to another person in various online social situations (see examples in Table 1). The scenarios were selected so that each item instantiates one practical goal within the same online text-only medium while holding the beneficiary and relationship constant. The language condition manipulates psychological dynamics; the lie/truth version manipulates the speaker’s practical goal; and the norm question manipulates the communication convention (descriptive vs. subjective). Thus, each trial represents a specific intersection of the three CCOLD dimensions. We will also add a mapping table indicating which scenario corresponds to each practical goal and which rating question corresponds to each norm dimension (see Table 2).

**Table 1:** Scenario examples across experiments.

Experiment	Category	Full scenario
Experiment 1	Black Lie	A and B met on an online platform and became a couple but have not yet met offline. B is secretly flirting with someone else on Xiaohongshu. A becomes suspicious and questions B via WeChat voice about whether B has been unfaithful. A has no evidence, and B tells A that he/she is not involved with a third person.
	White Lie	A and B are friends. A shares photos of homemade dishes on TikTok and @ B. B does not like the food but comments “So delicious! Best meal ever!” with a heart emoji to avoid hurting A’s feelings.
Experiment 2	White Lie	(Scenario Background): A and B are friendly coworkers. A’s mistake caused a project issue. (Outcome 1) B knows it was A’s fault. Although the boss is unable to verify who made the mistake, when the boss asks, B says that he/she does not know who made the mistake.
	Truth	(Scenario Background): A and B are friendly coworkers. A’s mistake caused a project issue. (Outcome 2) B knows it is A’s fault. The boss is unable to verify who made the mistake; when the boss asks, B says that it was A’s fault.
Experiment 3	Black Lie	(Scenario Background): A and B are friends. A messages B on QQ, requesting help promoting a Weibo post. B does not want to help. (Outcome 1) But B feels awkward refusing outright, and A is unaware of this. Therefore, B replies that he/she could not use the social media apps because of a phone problem.
	Truth	(Scenario Background): A and B are friends. A messages B on QQ, requesting help promoting a Weibo post. B does not want to help. (Outcome 2) So B responds that he/she does not want to help promote a Weibo post.

**Table 2:** CCOLD mapping across experiments.

CCOLD dimension	Operationalization	Experiment 1	Experiment 2	Experiment 3
Psychological dynamics	Language condition	Native Chinese vs. Foreign English	Native Chinese vs. Foreign English	Native Chinese vs. Foreign English
Practical goals	Lie/truth type	Black lie vs. White lie	White lie vs. Truth	Black lie vs. Truth
Communication conventions	Social norm question	Descriptive norm vs. Subjective norm; third-person likelihood vs. first-person willingness	Descriptive norm vs. Subjective norm; third-person likelihood vs. first-person willingness	Descriptive norm vs. Subjective norm; third-person likelihood vs. first-person willingness
Online medium	Text-only online platform	WeChat voice; TikTok; Xiaohongshu	Text-based online workplace scenario	QQ; Weibo

To ensure structural similarity and overall length of the original scenarios, the research team made slight modifications to the Chinese and English wording of the scenarios. Next, these scenarios were proofread by a native Chinese speaker and then translated into English by a bilingual Chinese-English researcher. The protagonists of the scenarios were named with capital letters, e.g., A and B; all adjectives and pronouns in the scenarios were gender-inclusive.

#### 3.1.4 Procedure

All participants completed the experiment online. Participants consented to the experiment after reading the description. They were then randomly assigned to complete the experimental tasks in Chinese (native language) or English (foreign language). Each participant read Chinese instructions and completed two practice trials before the main task (comprising 20 trials). In the two practice trials, participants were assigned to either the Chinese or English language group. Each trial consisted of a written scenario presented in the center of the screen (black font, white background). Participants were asked to rate on a Likert scale the likelihood of other people telling a lie (descriptive norm), from 1 (very unlikely) to 8 (very likely); simultaneously, participants were asked to rate from a first-person perspective their willingness to tell a lie (subjective norm), from 1 (very unwilling) to 8 (very willing). The scale appeared below each scenario. Participants clicked “Next Page” at the bottom of the screen to proceed to the next trial. Finally, they completed a demographic (including age, gender, immigration background, overseas living experience) and language background questionnaire (proficiency in FL and exposure to FL were rated on a 1–11 scale). In Experiment 1, self-reported questionnaire time was not recorded; platform-recorded time was not available.

#### 3.1.5 Analysis and results

Independent-samples *t*-tests compared the native- and foreign-language groups. In Experiment 1, self-reported proficiency in FL differed significantly (native *M* = 7.217, *SD* = 1.967; foreign *M* = 8.283, *SD* = 2.156), t(118) = −2.832, *p* = 0.005; and exposure to FL did not differ significantly (native *M* = 7.283, *SD* = 2.148; foreign *M* = 7.950, *SD* = 2.332), t(118) = −1.629, *p* = 0.106 (see Table 3). Because self-reported proficiency in FL differed significantly between language groups in Experiment 1, proficiency was included as a covariate in the final models for Experiment 1. The final models also controlled for age, gender, immigration background, and overseas living experience.

**Table 3:** Language background comparisons between the native- and foreign-language groups across experiments.

Experiment	Group	Age	Proficiency in FL	Exposure to FL	Age of acquisition (FL)
Experiment 1	Native language	21.083 (1.197)	7.22 (1.967)	7.28 (2.148)	——
	Foreign language	21.017 (1.444)	8.28 (2.156)	7.95 (2.332)	——
Experiment 2 and 3	Native language	21.150 (1.582)	62.817 (18.161)	54.483 (20.300)	6.233 (2.212)
	Foreign language	20.933 (1.666)	63.500 (15.613)	58.183 (20.844)	6.200 (2.305)

To investigate the effects of language type, lie type, and social norm type on cyber deception behavior, we performed a linear mixed-effects regression analysis. The model included fixed effects of language type (between-subjects variable: native vs. foreign), lie type (within-subjects variable: black vs. white), social norm type (within-subjects variable: descriptive norm vs. subjective norm), and the interactions among these factors (see Figure 1, descriptive statistics see Table 4).

**Figure 1:**
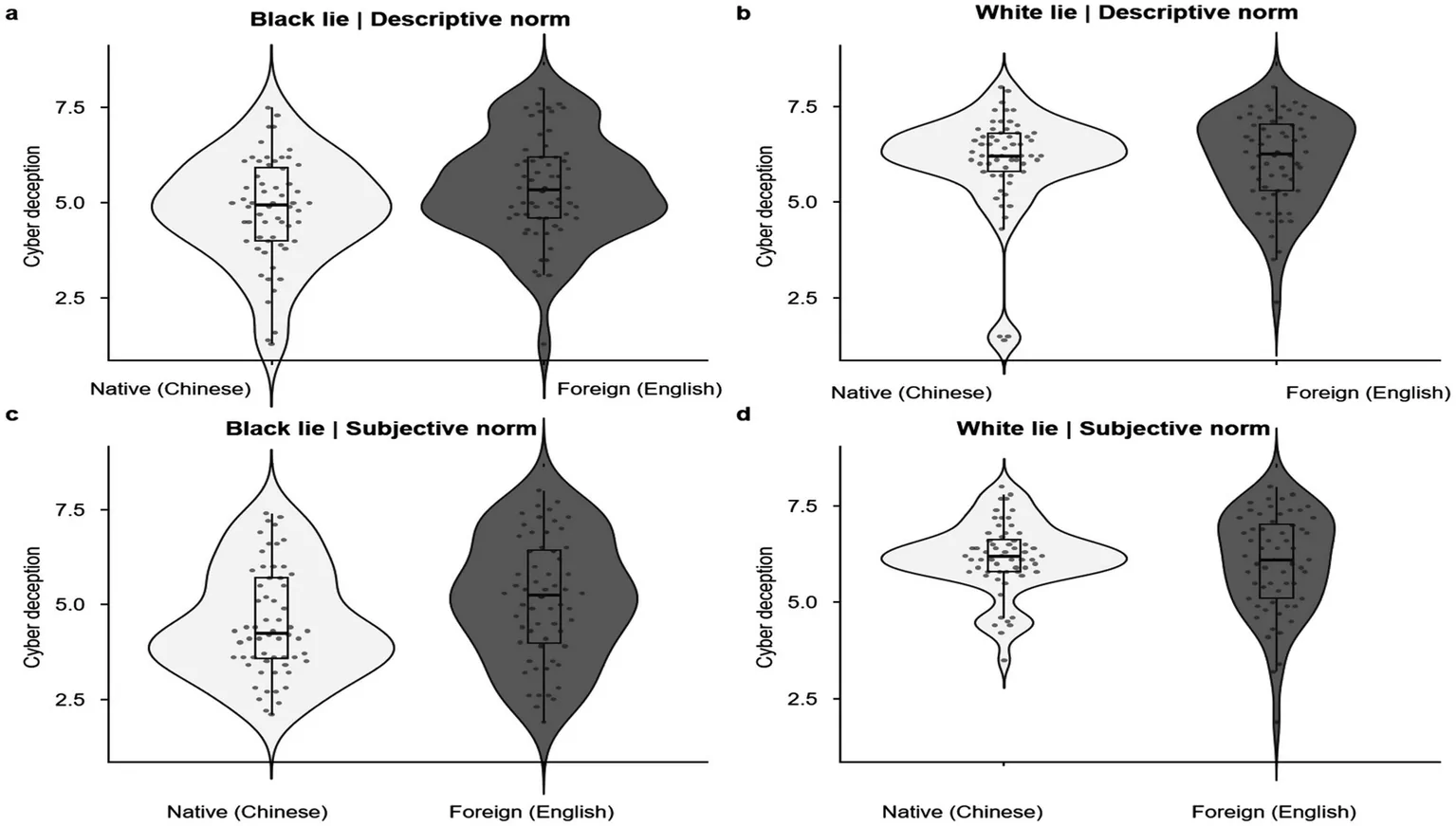
Acceptability of black and white lie-telling in native and foreign languages across descriptive and subjective norms. **(a)** shows black lies under the descriptive norm, **(b)** shows white lies under the descriptive norm, **(c)** shows black lies under the subjective norm, and **(d)** shows white lies under the subjective norm. Within each panel, thenative (Chinese) and foreign (English) language conditions are compared. Violin plots show the distribution of individual observations; box plots show the median, interquartile range, and 1.5 × IQR whiskers; jittered points represent individual raw data.

**Table 4:** Experiment 1 descriptive statistics.

Language type	Lie type	Social norm type	M	SD	*n*
Chinese	Black lie	Descriptive norm	4.82	1.38	60
Chinese	Black lie	Subjective norm	4.52	1.42	60
Chinese	White lie	Descriptive norm	6.08	1.31	60
Chinese	White lie	Subjective norm	6.16	0.91	60
English	Black lie	Descriptive norm	5.39	1.35	60
English	Black lie	Subjective norm	5.14	1.60	60
English	White lie	Descriptive norm	6.08	1.19	60
English	White lie	Subjective norm	6.02	1.28	60

The main effect of language type did not reach statistical significance (*b* = 0.434, *SE* = 0.238, *t*(467) = 1.827, *p* = 0.068). The main effect of lie type was significant (*b* = 1.263, *SE* = 0.185, *t*(467) = 6.844, *p* < 0.001), indicating that the acceptability of white lies was higher than that of black lies. The main effect of social norm type was not significant (*b* = −0.297, *SE* = 0.185, *t*(467) = −1.607, *p* = 0.109).

The language × lie interaction was significant (*b* = −0.580, *SE* = 0.261, *t*(467) = −2.222, *p* = 0.027). The language × norm interaction was not significant (*b* = 0.038, *SE* = 0.261, *t*(467) = 0.147, *p* = 0.883), and the lie × norm interaction was not significant (*b* = 0.380, *SE* = 0.261, *t*(467) = 1.456, *p* = 0.146). The three-way interaction was not significant (*b* = −0.180, *SE* = 0.369, *t*(467) = −0.488, *p* = 0.626).

For the language × lie interaction, within black lies, the foreign-language condition (*M* = 4.67, *SE* = 0.480) was rated significantly higher than the native-language condition (*M* = 4.22, *SE* = 0.499), *b* = −0.453, *SE* = 0.199, *t*(80) = −2.283, *p* = 0.025. Within white lies, the native-language condition (*M* = 5.68, *SE* = 0.499) and the foreign-language condition (*M* = 5.46, *SE* = 0.480) did not differ significantly, *b* = 0.217, *SE* = 0.199, *t*(80) = 1.091, *p* = 0.279. Within the native language, white lies (*M* = 5.68, *SE* = 0.484) were rated significantly higher than black lies (*M* = 4.22, *SE* = 0.484), *b* = −1.453, *SE* = 0.131, *t*(24.1) = −11.135, *p* < 0.001. Within the foreign language, white lies (*M* = 5.46, *SE* = 0.484) were also rated significantly higher than black lies (*M* = 4.67, *SE* = 0.484), *b* = −0.783, *SE* = 0.131, *t*(24.1) = −6.001, *p* < 0.001.

The covariates of age (*b* = 0.078, *SE* = 0.067, *t*(467) = 1.177, *p* = 0.240), gender (*b* = −0.057, *SE* = 0.171, *t*(467) = −0.333, *p* = 0.740), immigration background (*b* = 0.777, *SE* = 0.977, *t*(467) = 0.796, *p* = 0.427), and overseas living experience (*b* = 0.366, *SE* = 0.277, *t*(467) = 1.322, *p* = 0.187) were not significant. Self-reported English proficiency was significantly associated with acceptability (*b* = 0.172, *SE* = 0.041, *t*(467) = 4.147, *p* < 0.001).

These findings partly support Hypothesis 1. Specifically, the results support Hypothesis 1a and 1b, but deny Hypothesis 1c and 1d. Therefore, Experiment 1 found that foreign language use significantly increased the acceptability of black lies, but there was no significant change in the evaluation of white lies. White lies, due to their altruistic nature, may be perceived as socially acceptable behavior across different linguistic environments, meaning no difference was observed between native and foreign language contexts.

### 3.2 Experiment 2: how second language influences cyber deception: white lies and truth

#### 3.2.1 Research purpose

The intrinsic difference between black and white lies, apart from different emotional values, may also be due to the fact that the two kinds of lies are fundamentally different scenarios. To further examine whether foreign language effects exist under the same scenario and the regulatory role of social norms. There have been studies involving only the comparison between white lies and the truth (Hu and Navarrete, 2024), however, the results of Experiment 1 indicate that the reduction effect of foreign languages on social norms occurs only in black lies rather than white lies, therefore, we should be more hopeful to see a comparison between white lies and the truth in the same network scenario. Furthermore, Hu and Navarrete (2024) use the level of acceptance of lies when screening for white lies, and the researchers consider that lies with high levels of acceptance are white lies, this screening condition is also inappropriate. Normally, we think that white lies should be well-intentioned lies, while black lies are not (Lu et al., 2019), so it is not easy to judge black and white lies simply from their acceptability. Therefore, an improvement was made: Before experiment 2 began, a screening of experimental material from experiment 1 was conducted. To select white lies, we extracted data from all native-language lie scenarios, treating the 6 most acceptable or most willing lies out of 10 white lies as white lies, and treating the 6 least acceptable or least willing lies out of 10 black lies as black lies. Also, the accuracy of distinguishing responses and sensitivity to subtle changes in participants’ opinions when measuring dependent variables using the 8-point Likert scale may be limited. To overcome these limitations, we adopted a 0–100 score in subsequent Experiment 2 and 3.

Experiment 2 attempts to explore how second language, white lie status (white lie vs. truth), and social norm type influence individual cyber deception behavior. Experiment 2 explores the FLE of cyber deception and examines the roles of lie type (white lie/truth) and social norm (descriptive norm/subjective norm). This study used a mixed experimental design; participants were randomly assigned to either a native (Chinese) or a foreign (English) condition and were asked to assess the likelihood of deception occurring in cyber deception scenarios from a third-person or first-person perspective.

#### 3.2.2 Participants

This study used *a priori* power analysis in G*Power (Faul et al., 2007) to estimate the sample size, selecting a small to medium effect size f^2^ = 0.02 (Cantarero et al., 2018; Hu and Navarrete, 2024), α = 0.05, and power = 0.95, yielding a required sample size of 108. Therefore, 120 participants (*N* = 120, 55 males and 65 females) were planned to be recruited from an online platform, with 60 participants randomly assigned to the native language group and the other 60 participants randomly assigned to the English group. The average age of participants was 21.04 years old (SD = 1.621, range = 18–26).

#### 3.2.3 Materials

We selected 6 white lie scenarios and 6 corresponding truth scenarios from Experiment 1 (Cantarero et al., 2018; Hu and Navarrete, 2024; Moreno et al., 2016). In these scenarios, a person lies or tells the truth to another person in various online social situations (see examples in Table 1). We will also add a mapping table indicating which scenario corresponds to each practical goal and which rating question corresponds to each norm dimension in this experiment (see Table 2).

To ensure structural similarity and overall length of the original scenarios, the research team made slight modifications to the Chinese and English wording of the scenarios. Next, these scenarios were proofread by a native Chinese speaker and then translated into English by a bilingual Chinese-English researcher. The protagonists of the scenarios were named with capital letters, e.g., A and B; all adjectives and pronouns in the scenarios were gender-inclusive.

Experiment 1 used an 8-point Likert scale, consistent with Hu and Navarrete (2024), whereas Experiments 2 and 3 used a 0–100 slider scale to increase sensitivity for truth versus lie comparisons. Because each experiment was analyzed separately with linear mixed-effects models and raw scores were not pooled, the different scales did not require statistical transformation. Cross-experiment conclusions were based on the direction and significance of effects rather than on direct numerical comparison.

#### 3.2.4 Procedure

All participants completed the experiment on an online survey platform. Participants consented to the experiment after reading the description. They were then randomly assigned to complete the experimental tasks in Chinese (native language) or English (foreign language). Each participant read Chinese instructions and completed a language comprehension task before the formal task (comprising 6 trials). The language comprehension task was presented in Chinese for the native language group and in English for the foreign language group. They completed a demographic and language background questionnaire [proficiency, exposure, and age of acquisition (FL) were assessed before the experimental tasks on 0–100 scales].

In the formal task, each trial consisted of a written scenario presented in the center of the screen (black font, white background). Participants were asked to rate on a Likert scale the likelihood of other people telling a lie (descriptive norm), from 0 (completely impossible) to 100 (completely possible); simultaneously, participants were asked to rate from a first-person perspective their willingness to tell a lie (subjective norm), from 0 (completely unwilling) to 100 (completely willing). The scale appeared below each scenario. Also, participants were asked to rate on a Likert scale the likelihood of other people telling the truth (descriptive norm), from 0 (completely impossible) to 100 (completely possible); simultaneously, participants were asked to rate from a first-person perspective their willingness to tell the truth (subjective norm), from 0 (completely unwilling) to 100 (completely willing). The scale appeared below each scenario. Participants clicked “Next Page” at the bottom of the screen to proceed to the next trial. In Experiment 2, self-reported questionnaire time, *M* = 7.50 min, *SD* = 5.05 min; Median = 6 min, IQR = 5–9 min.

#### 3.2.5 Analysis and results

Independent-samples *t*-tests compared the native- and foreign-language groups. Experiments 2 used the same pre-experiment baseline data. In these experiments, self-reported proficiency did not differ significantly (native *M* = 62.817, *SD* = 18.161; foreign *M* = 63.500, *SD* = 15.613), *t*(118) = −0.221, *p* = 0.825; and exposure to FL did not differ significantly (native *M* = 54.483, *SD* = 20.300; foreign *M* = 58.183, *SD* = 20.844), *t*(118) = −0.985, *p* = 0.327; age of acquisition (FL) did not differ significantly (native *M* = 6.233, *SD* = 2.212; foreign *M* = 6.200, *SD* = 2.305), *t*(118) = 0.081, *p* = 0.936 (see Table 3). In Experiments 2, the language groups did not differ significantly in proficiency, exposure, and age of acquisition (FL), but proficiency was retained as a covariate for consistency; exposure was examined descriptively and was not entered into the primary models. The final models also controlled for age, gender, immigration background, and overseas living experience.

To investigate the effects of language type, white lie type, and social norm type on cyber deception behavior, we performed a linear mixed-effects regression analysis. The model included fixed effects of language type (between-subjects variable: native vs. foreign), white lie status (within-subjects variable: white lie vs. truth), social norm type (within-subjects variable: descriptive norm vs. subjective norm), and the interactions among these factors (see Figure 2, descriptive statistics see Table 5).

**Figure 2:**
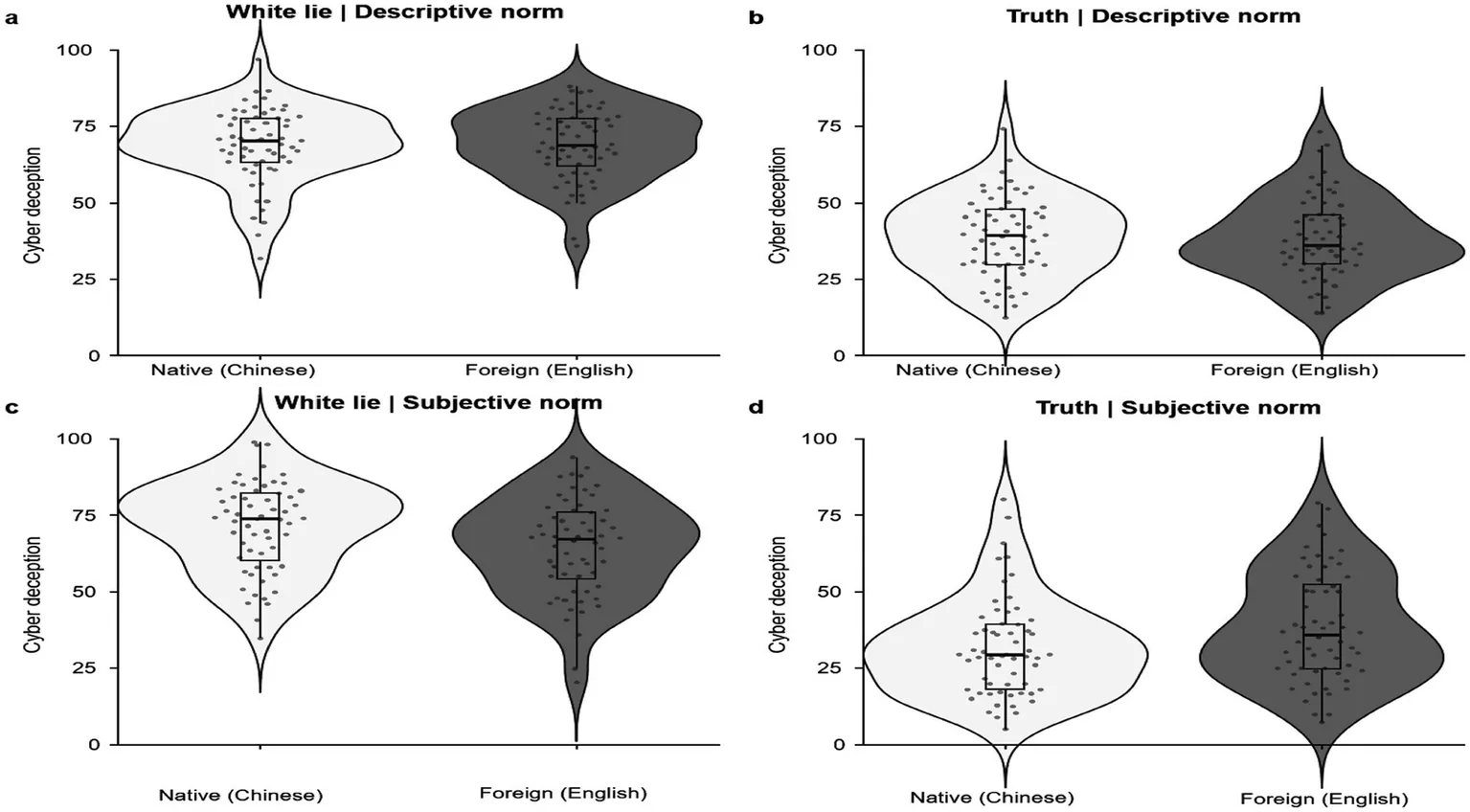
Acceptability of white lie-telling and truth-telling in native and foreign languages across descriptive and subjective norms. **(a)** shows white lies under the descriptive norm, **(b)** shows truth under the descriptive norm, **(c)** shows white lies under the subjective norm, and **(d)** shows truth under the subjective norm. Within each panel, the native (Chinese) and foreign (English) language conditions are compared. Violin plots show the distribution of individual observations; box plots show the median, interquartile range, and 1.5 × IQR whiskers; jittered points represent individual raw data.

**Table 5:** Experiment 2 descriptive statistics.

Language type	Lie/truth type	Social norm type	M	SD	*n*
Chinese	White lie	Descriptive norm	68.93	12.31	60
Chinese	White lie	Subjective norm	71.34	14.84	60
Chinese	Truth	Descriptive norm	38.85	13.21	60
Chinese	Truth	Subjective norm	31.52	16.18	60
English	White lie	Descriptive norm	68.98	11.72	60
English	White lie	Subjective norm	64.32	16.06	60
English	Truth	Descriptive norm	38.45	13.45	60
English	Truth	Subjective norm	38.36	17.99	60

The main effect of language type was not significant (*b* = −0.162, *SE* = 2.677, *t*(467) = −0.060, *p* = 0.952). The main effect of lie/truth type was significant (*b* = −30.075, *SE* = 2.846, *t*(467) = −10.569, *p* < 0.001), indicating that truth was rated lower than white lies. The main effect of social norm type was not significant (*b* = 2.417, *SE* = 2.846, *t*(467) = 0.849, *p* = 0.396).

The language × lie/truth interaction was not significant (*b* = −0.458, *SE* = 4.024, *t*(467) = −0.114, *p* = 0.909). The language × norm interaction was not significant (*b* = −7.075, *SE* = 4.024, *t*(467) = −1.758, *p* = 0.079). The lie/truth × norm interaction was significant (*b* = −9.747, *SE* = 4.024, *t*(467) = −2.422, *p* = 0.016), and the three-way interaction was significant (*b* = 14.319, *SE* = 5.691, *t*(467) = 2.516, *p* = 0.012).

For the lie/truth × norm interaction, white lies were rated significantly higher than truth under both the descriptive norm (White lie *M* = 65.0, *SE* = 3.15; Truth *M* = 34.7, *SE* = 3.15; *b* = 30.3, *SE* = 2.01, *t*(277) = 15.061, *p* < 0.001) and the subjective norm (White lie *M* = 63.8, *SE* = 3.15; Truth *M* = 31.0, *SE* = 3.15; *b* = 32.9, *SE* = 2.01, *t*(277) = 16.347, *p* < 0.001). Norm did not differ significantly within white lies (Descriptive *M* = 65.0, *SE* = 3.15; Subjective *M* = 63.8, *SE* = 3.15; *b* = 1.12, *SE* = 2.01, *t*(277) = 0.557, *p* = 0.578) or truth (Descriptive *M* = 34.7, *SE* = 3.15; Subjective *M* = 31.0, *SE* = 3.15; *b* = 3.71, *SE* = 2.01, *t*(277) = 1.843, *p* = 0.066).

For the three-way interaction, language did not differ significantly under the descriptive norm for white lies (Native *M* = 65.0, *SE* = 3.38; Foreign *M* = 64.9, *SE* = 3.47; *b* = 0.162, *SE* = 2.68, *t*(241) = 0.060, *p* = 0.952) or truth (Native *M* = 35.0, *SE* = 3.38; Foreign *M* = 34.4, *SE* = 3.47; *b* = 0.620, *SE* = 2.68, *t*(241) = 0.232, *p* = 0.817). Under the subjective norm, language differed significantly for white lies (Native *M* = 67.5, *SE* = 3.38; Foreign *M* = 60.2, *SE* = 3.47; *b* = 7.237, *SE* = 2.68, *t*(241) = 2.704, *p* = 0.007) and for truth (Native *M* = 27.6, *SE* = 3.38; Foreign *M* = 34.3, *SE* = 3.47; *b* = −6.624, *SE* = 2.68, *t*(241) = −2.475, *p* = 0.014).

The covariates of age, gender, immigration background, overseas living experience, and self-reported English proficiency did not significantly predict acceptability, all ps > 0.05.

These findings partly support Hypothesis 2. Specifically, the results support Hypothesis 2a, but deny Hypothesis 2b, 2c and 2d. Therefore, Experiment 2 found that when telling a white lie oneself, willingness was higher in the Chinese condition than in the foreign language condition; when telling the truth oneself, willingness was higher in the foreign language condition than in the Chinese condition.

### 3.3 Experiment 3: how second language influences cyber deception: black lies and truth

#### 3.3.1 Research purpose

This study attempts to explore how second language, black lie status (black lie vs. truth), and social norm type influence individual cyber deception behavior. Experiment 3 explores the FLE of cyber deception and examines the role of lie type (black lie/truth) and social norm (descriptive norm/subjective norm). This study used a mixed experimental design; participants were randomly assigned to either a native (Chinese) or a foreign (English) condition and were asked to assess the likelihood of deception occurring in cyber deception scenarios from a third-person or first-person perspective.

#### 3.3.2 Participants

This study used *a priori* power analysis in G*Power (Faul et al., 2007) to estimate the sample size, selecting a small effect size f^2^ = 0.02 (Cantarero et al., 2018; Hu and Navarrete, 2024), α = 0.05, and power = 0.95, yielding a required sample size of 108. In 120 participants which were recruited in Experiment 2, 60 participants randomly assigned to the native language group and the other 60 participants randomly assigned to the English group.

#### 3.3.3 Materials

We selected six black lie scenarios and six corresponding truth scenarios from Experiment 1 (Cantarero et al., 2018; Hu and Navarrete, 2024; Moreno et al., 2016). In these scenarios, a person lies or tells the truth to another person in various online social situations (see examples in Table 1). We will also add a mapping table indicating which scenario corresponds to each practical goal and which rating question corresponds to each norm dimension in this experiment (see Table 2).

To ensure structural similarity and overall length of the original scenarios, the research team made slight modifications to the Chinese and English wording of the scenarios. Next, these scenarios were proofread by a native Chinese speaker and then translated into English by a bilingual Chinese-English researcher. The protagonists of the scenarios were named with capital letters, e.g., A and B; all adjectives and pronouns in the scenarios were gender-inclusive.

#### 3.3.4 Procedure

All participants completed the experiment on an online survey platform. Participants consented to the experiment after reading the description. They were then randomly assigned to complete the experimental tasks in Chinese (native language) or English (foreign language). Each participant read Chinese instructions and completed a language comprehension task before the formal task (comprising 6 trials). The language comprehension task was presented in Chinese for the native language group and in English for the foreign language group. They completed a demographic and language background questionnaire (proficiency, exposure, and age of acquisition (FL) were assessed before the experimental tasks on 0–100 scales).

In the formal task, each trial consisted of a written scenario presented in the center of the screen (black font, white background). Participants were asked to rate on a Likert scale the likelihood of other people telling a lie (descriptive norm), from 0 (completely impossible) to 100 (completely possible); simultaneously, participants were asked to rate from a first-person perspective their willingness to tell a lie (subjective norm), from 0 (completely unwilling) to 100 (completely willing). The scale appeared below each scenario. Also, participants were asked to rate on a Likert scale the likelihood of other people telling the truth (descriptive norm), from 0 (completely impossible) to 100 (completely possible); simultaneously, participants were asked to rate from a first-person perspective their willingness to tell the truth (subjective norm), from 0 (completely unwilling) to 100 (completely willing). The scale appeared below each scenario. Participants clicked “Next Page” at the bottom of the screen to proceed to the next trial. In Experiment 3, self-reported questionnaire time, M = 4.87 min, SD = 2.99 min; Median = 4 min, IQR = 3–6 min.

#### 3.3.5 Analysis and results

Like Experiment 2, the final model of Experiment 3 controlled for age, gender, proficiency, immigration background, and overseas living experience.

To investigate the effects of language type, black lie status, and social norm type on cyber deception behavior, we performed a linear mixed-effects regression analysis. The model included fixed effects of language type (between-subjects variable: native vs. foreign), black lie status (within-subjects variable: black lie vs. truth), social norm type (within-subjects variable: descriptive norm vs. subjective norm), and the interactions among these factors (see Figure 3, descriptive statistics see Table 6).

**Figure 3:**
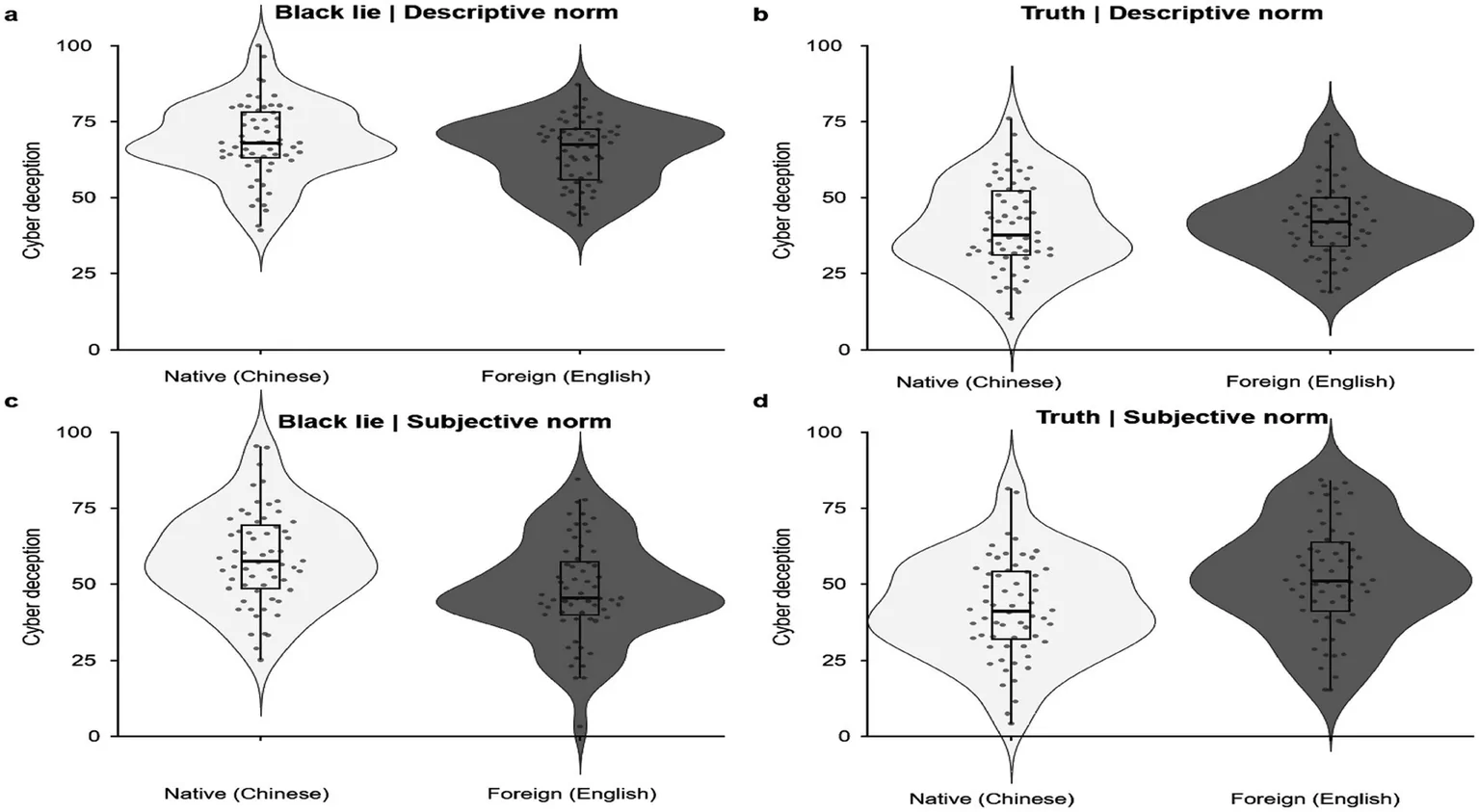
Acceptability of black lie-telling and truth-telling in native and foreign languages across descriptive and subjective norms. **(a)** shows black lies under the descriptive norm, **(b)** shows truth under the descriptive norm, **(c)** shows black lies under the subjective norm, and **(d)** shows truth under the subjective norm. Within each panel, the native (Chinese) and foreign (English) language conditions are compared. Violin plots show the distribution of individual observations; box plots show the median, interquartile range, and 1.5 × IQR whiskers; jittered points represent individual raw data.

**Table 6:** Experiment 3 descriptive statistics.

Language type	Lie/truth type	Social norm type	M	SD	*n*
Chinese	Black lie	Descriptive norm	68.64	12.65	60
Chinese	Black lie	Subjective norm	58.77	15.69	60
Chinese	Truth	Descriptive norm	40.36	14.60	60
Chinese	Truth	Subjective norm	41.90	16.39	60
English	Black lie	Descriptive norm	64.83	10.97	60
English	Black lie	Subjective norm	48.14	16.30	60
English	Truth	Descriptive norm	42.34	12.58	60
English	Truth	Subjective norm	52.44	17.86	60

The main effect of language type was not significant (*b* = −4.016, *SE* = 2.705, *t*(467) = −1.485, *p* = 0.138). The main effect of lie/truth type was significant (*b* = −28.286, *SE* = 2.776, *t*(467) = −10.190, *p* < 0.001), indicating that truth was rated lower than black lies. The main effect of social norm type was significant (*b* = −9.872, *SE* = 2.776, *t*(467) = −3.556, *p* < 0.001), indicating that the descriptive norm condition was rated significantly higher than the subjective norm condition.

The language × lie/truth interaction was not significant (*b* = 5.803, *SE* = 3.926, *t*(467) = 1.478, *p* = 0.140). The language × norm interaction was not significant (*b* = −6.811, *SE* = 3.926, *t*(467) = −1.735, *p* = 0.083). The lie/truth × norm interaction was significant (*b* = 11.417, *SE* = 3.926, *t*(467) = 2.908, *p* = 0.004), and the three-way interaction was significant (*b* = 15.361, *SE* = 5.552, *t*(467) = 2.767, *p* = 0.006).

For the lie/truth × norm interaction, black lies were rated significantly higher than truth under both the descriptive norm (Black lie *M* = 67.7, *SE* = 3.65; Truth *M* = 42.3, *SE* = 3.65; *b* = 25.38, *SE* = 1.96, *t*(164) = 12.932, *p* < 0.001) and the subjective norm (Black lie *M* = 54.4, *SE* = 3.65; Truth *M* = 48.1, *SE* = 3.65; *b* = 6.29, *SE* = 1.96, *t*(164) = 3.203, *p* = 0.002). Norm differed significantly within black lies (Descriptive *M* = 67.7, *SE* = 3.65; Subjective *M* = 54.4, *SE* = 3.65; *b* = 13.28, *SE* = 1.96, *t*(164) = 6.764, *p* < 0.001) and truth (Descriptive *M* = 42.3, *SE* = 3.65; Subjective *M* = 48.1, *SE* = 3.65; *b* = −5.82, *SE* = 1.96, *t*(164) = −2.965, *p* = 0.004).

For the three-way interaction, language did not differ significantly under the descriptive norm for black lies (Native *M* = 69.7, *SE* = 3.84; Foreign *M* = 65.7, *SE* = 3.94; *b* = 4.02, *SE* = 2.71, *t*(152) = 1.485, *p* = 0.140) or truth (Native *M* = 41.4, *SE* = 3.84; Foreign *M* = 43.2, *SE* = 3.94; *b* = −1.79, *SE* = 2.71, *t*(152) = −0.660, *p* = 0.510). Under the subjective norm, language differed significantly for black lies (Native *M* = 59.8, *SE* = 3.84; Foreign *M* = 49.0, *SE* = 3.94; *b* = 10.83, *SE* = 2.71, *t*(152) = 4.002, *p* < 0.001) and for truth (Native *M* = 43.0, *SE* = 3.84; Foreign *M* = 53.3, *SE* = 3.94; *b* = −10.34, *SE* = 2.71, *t*(152) = −3.821, *p* = 0.0002).

The covariates of age, gender, immigration background, overseas living experience, and self-reported English proficiency did not significantly predict acceptability, all ps > 0.05.

These findings partly support Hypothesis 3. Specifically, the results support Hypothesis 3a, but deny Hypothesis 3b, 3c and 3d. Therefore, Experiment 3 found that when telling a black lie oneself, willingness was significantly higher in the Chinese condition than in the English condition; when telling the truth oneself, willingness was significantly higher in the English condition than in the Chinese condition.

## 4 Discussion

Through three systematic experiments, this study deeply investigates the influence of second language use (i.e., the foreign language effect) on the psychological mechanism of cyber deception among university students within the framework of the CCOLD. The study focuses not only on the core variable of language switching but also meticulously distinguishes the roles of lie type (black lies/white lies/truth) and social norms (descriptive norm/subjective norm), thereby providing a multidimensional perspective on the complexity of moral decision-making in online environments.

The results of Experiment 1 are consistent with Hu and Navarrete (2024) and Gai and Puntoni (2021), showing that foreign language use significantly increased the acceptability of black lies. However, unlike Hu and Navarrete (2024), whose finding was mainly observed under descriptive norms, the present study’s finding did not show a difference between descriptive and subjective norms. Additionally, Gai and Puntoni (2021) argued that a foreign language reduces white lies (benevolent lies), while the present study found no effect of foreign language on white lies.

The results of Experiment 2 are consistent with Hu and Navarrete (2024) and Gai and Puntoni (2021) in that, when telling a white lie, willingness was higher in the Chinese condition than in the foreign language condition; when telling the truth, willingness was higher in the foreign language condition than in the Chinese condition. However, inconsistently, this finding primarily focused on subjective norms rather than descriptive norms, contrary to Hu and Navarrete (2024).

Experiment 3 found that when telling a black lie, willingness was significantly higher in the Chinese condition than in the English condition; when telling the truth, willingness was significantly higher in the English condition than in the Chinese condition. Consistent with Bereby-Meyer et al. (2020), who found across four countries (Israel, South Korea, Spain, USA) using a dice-rolling task that participants in native language conditions were more inclined to falsely report higher numbers for profit, while participants in foreign language conditions were more honest (report distribution closer to random). That is, Foreign language reduced black lies. Furthermore, consistent with Suchotzki and Gamer (2018), who found that a foreign language prolonged reaction times for truth-telling, mainly interfering with truth-telling rather than promoting lying. Thus, the finding of Experiment 3 challenges the view that foreign language makes lying easier.

However, the finding above of Experiment 3 is inconsistent with existing studies (Alempaki et al., 2021; Gai and Puntoni, 2021; Hu and Navarrete, 2024; Suchotzki and Gamer, 2018). These results are inconsistent with Hu and Navarrete (2024) and Alempaki et al. (2021) in that this finding to some extent denies the “norm-weakening phenomenon of foreign language; norm-strictness phenomenon of native language.” Also inconsistently, this finding primarily focused on subjective norms rather than descriptive norms (Hu and Navarrete, 2024; Alempaki et al., 2021). Regarding the differences in norms, neither Hu and Navarrete (2024) nor Alempaki et al. (2021) placed descriptive norms (social norms) and subjective norms (personal norms) within one single study. Therefore, this study attempts to explore different norms within the same study to clarify the comparison between them more explicitly, which is an important innovative point of this research. Moreover, inconsistent with Gai and Puntoni (2021), who argued that a foreign language increases black lies (self-interested lies) by reducing the self-diagnosticity of behavior.

Overall, except for Experiment 1, Experiment 2 and 3 both denies that norm-weakening phenomenon of foreign language and the norm-strictness phenomenon of native language (such as Hu and Navarrete, 2024; Alempaki et al., 2021). In other words, foreign languages do not increase the acceptance of lies, whether black lies or white lies; instead, foreign languages increase the acceptance of the truth.

In any case, this study cannot support the absence of a foreign language effect (Duñabeitia and Costa, 2015). We speculate that this is due to the substantial differences in experimental materials. Duñabeitia and Costa (2015) required participants to truthfully or falsely describe animal pictures without monetary or material rewards, nor motives to protect others, which constitutes “experimental lies” rather than black or white lies. The absence of motivation may lead to a null result between foreign language and deceptive behavior, i.e., no foreign language effect. After all, previous studies (Hu and Navarrete, 2024; Alempaki et al., 2021; Gai and Puntoni, 2021; Suchotzki and Gamer, 2018; Duñabeitia and Costa, 2015; Bereby-Meyer et al., 2020) and the present study all employed materials involving black lies or white lies.

Why black and white lies differ in cyber deception? Experiment 1 showed that white lies were evaluated as significantly more acceptable than black lies. Foreign language use increased black lie acceptability but did not significantly alter white lie acceptability. Black lies are self-serving and are generally seen as violations of moral and relational trust (Lu et al., 2019); their acceptability is therefore more sensitive to emotional and normative cues, and foreign language use may dampen that sensitivity under subjective norms (Bereby-Meyer et al., 2020; Hu and Navarrete, 2024). White lies are other-oriented and prosocial (Cantarero et al., 2018; Lu et al., 2019), but the present findings show that: In Experiment 2, foreign language use reduced white lie acceptability under subjective norms and increased truth acceptability, whereas in Experiment 3 it reduced black lie acceptability under subjective norms and increased truth acceptability. Thus, the moral distinction between black and white lies remains central, but its expression depends on whether the online context evokes descriptive or subjective norms.

### 4.1 Theoretical implications

Extension of the CCOLD and IDT. This study applied the CCOLD model to the specific behavior of cyber deception, empirically examining how context (linguistic context) interacts with behavior type (lie type) and cognitive norms. The findings perfectly illustrate the dynamic interactive nature of the model: cyber deceptive behavior is not determined by a single factor but results from the synergistic operation of linguistic cognitive mode, the inherent moral attribute of the communicative act, and the internalized social rules of the individual. For example, the evaluation of the likelihood of a black lie (high negative moral encoding) occurring undergoes a unique change under the superimposed context of a foreign language (low emotional involvement) and subjective norms (high self-relevance). This goes a step further than IDT, which merely emphasizes the influence of contextual changes on deception strategies, by concretizing context as linguistic context and quantifying its differential impact on the perception of deceptive behaviors with different motivations.

Modification Regarding Social Norms in the Online Context. The present study suggests that text-based online interaction may reshape the functioning of social norms. The findings show that language effects were not uniform across conditions; they emerged mainly under subjective norms and depended on lie/truth type. In Experiments 2 and 3, significant three-way interactions showed that, under subjective norms, foreign-language use increased truth acceptance while reducing black-lie and white-lie acceptance; under descriptive norms, the language effect was not significant. Thus, when evaluating first-person willingness to lie or tell the truth online, individuals may rely more strongly on subjective norms than on descriptive norms. This interpretation further specifies that this effect is conditional on both the moral nature of the communicative act and the norm dimension.

### 4.2 Limitations and future directions

Sample Representativeness: The participants were university students from China, making the group relatively homogeneous in age and educational background. Caution is needed when generalizing the findings to other age groups, educational levels, or cultural backgrounds, as different combinations of native and foreign languages (e.g., Chinese-German, Chinese-English) may yield different effects.

Specificity of Online Context: Although the research focused on online scenarios, it did not differentiate between the nuances of various online platforms (e.g., anonymous forums vs. real-name social software), nor did it control for social distance, relationship intimacy, and other variables in the online interpersonal domain, which are likely key factors.

Behavioral Experiments and Longitudinal Studies: Future research should develop experimental paradigms based on real interaction tasks to directly observe actual deceptive behavior during foreign language use. Additionally, the experience sampling method could be adopted to track individuals who regularly use a foreign language for online social interaction over a long period, studying the long-term changes in their deceptive behavior and perceptions.

Practically, the finding that foreign-language use did not uniformly increase lying, but instead produced condition-specific effects—increasing truth acceptance while reducing black-lie and white-lie acceptance under subjective norms—suggests that anti-fraud interventions should not assume bilingual users are more deceptive. Internet platform designers can (a) strengthen subjective norms by prompting users to reflect on their own ethical standards before posting; (b) reduce ambiguity in descriptive norms by showing transparent, verified online community norms.

The present study had related limitations concerning task duration and AI translation. First, the online platform did not automatically record task duration, and Experiment 1 did not include a self-reported questionnaire-time item; therefore, only Experiments 2 and 3 provided self-reported questionnaire time. Second, although participants were required to pass comprehension checks before beginning the tasks, we cannot fully rule out the possibility that participants in the foreign-language condition used unauthorized AI translation tools. Future studies should simultaneously collect self-reported questionnaire time and automatically logged platform duration, apply predetermined too-short and too-long time windows as quality checks, administer post-task comprehension checks, ask participants to self-report any use of AI translation tools, and experimentally manipulate access to AI translation as a moderator of foreign-language effects on cyber deception.

## Data Availability

The original contributions presented in the study are included in the article/supplementary material, further inquiries can be directed to the corresponding author.
